# Diversifying Doulas Initiative: Improving Maternal Health Outcomes in People of Color Through Doula Care

**DOI:** 10.1089/heq.2023.0082

**Published:** 2024-07-01

**Authors:** Sharee Livingston, Cherise Hamblin, Crista Johnson, LaShekia Chatman, Kayla Bolden

**Affiliations:** ^1^ Department of Obstetrics and Gynecology, University of Pittsburgh Medical Center, Lititz, Pennsylvania, USA.; ^2^ Lancaster General Hospital, Lancaster, Pennsylvania, USA.; ^3^ UMass Chan Medical School, Worcester, Massachusetts, USA.; ^4^ State University of New York at Buffalo, Buffalo, New York, USA.; ^5^ CDC, Atlanta, Georgia, USA.

**Keywords:** Dr. Cherise Hamblin, Dr. Crista Johnson-Agbakwu, LaShekia Chatman, Kayla Bolden

## Abstract

The Diversifying Doulas Initiative (DDI) aims to improve maternal health outcomes in Black and Brown people through doula care in Lancaster County. DDI trained 28 Black and Brown doulas and provided fully subsidized doula care to over 200 patients of color giving birth in Lancaster County. The perinatal workforce comprises community birth workers, doulas, midwives, nurses, students, and physicians. By diversifying the perinatal workforce and increasing access to doulas, patients of color benefit from a proven intervention.

## Introduction

Birthing people of color are disproportionately affected by the most common causes of maternal morbidity and mortality.^1^ While doula support during pregnancy, labor, and birth has been demonstrated to improve outcomes, people of color have less access to doula services than their White counterparts.^2^ Community-based doula programs like the Diversifying Doulas Initiative (DDI) work to increase access to doula services for people of color.

Doulas are trained professionals who assist pregnant people with physical, emotional, and informational support before, during, and after labor. Research shows that people receiving support from doulas have less postpartum depression and anxiety, less postpartum psychosis, lower cesarean section rates, less low birth weight newborns, and less chronic illnesses such as hypertension and diabetes.^3^

In July 2020, we created the DDI to mitigate any worsening maternal health disparities gaps that could result from the COVID-19 pandemic. Our DDI mission is to decrease maternal morbidity and mortality among pregnant people of color through doula care.

DDI aims to:

- Train people of color to become doulas
- Provide pregnant people of color with fully subsidized doula care
- Conduct research on Black and Brown maternal health outcomes
- Address the maternal mortality crisis in the United States

**FIG. 1.**
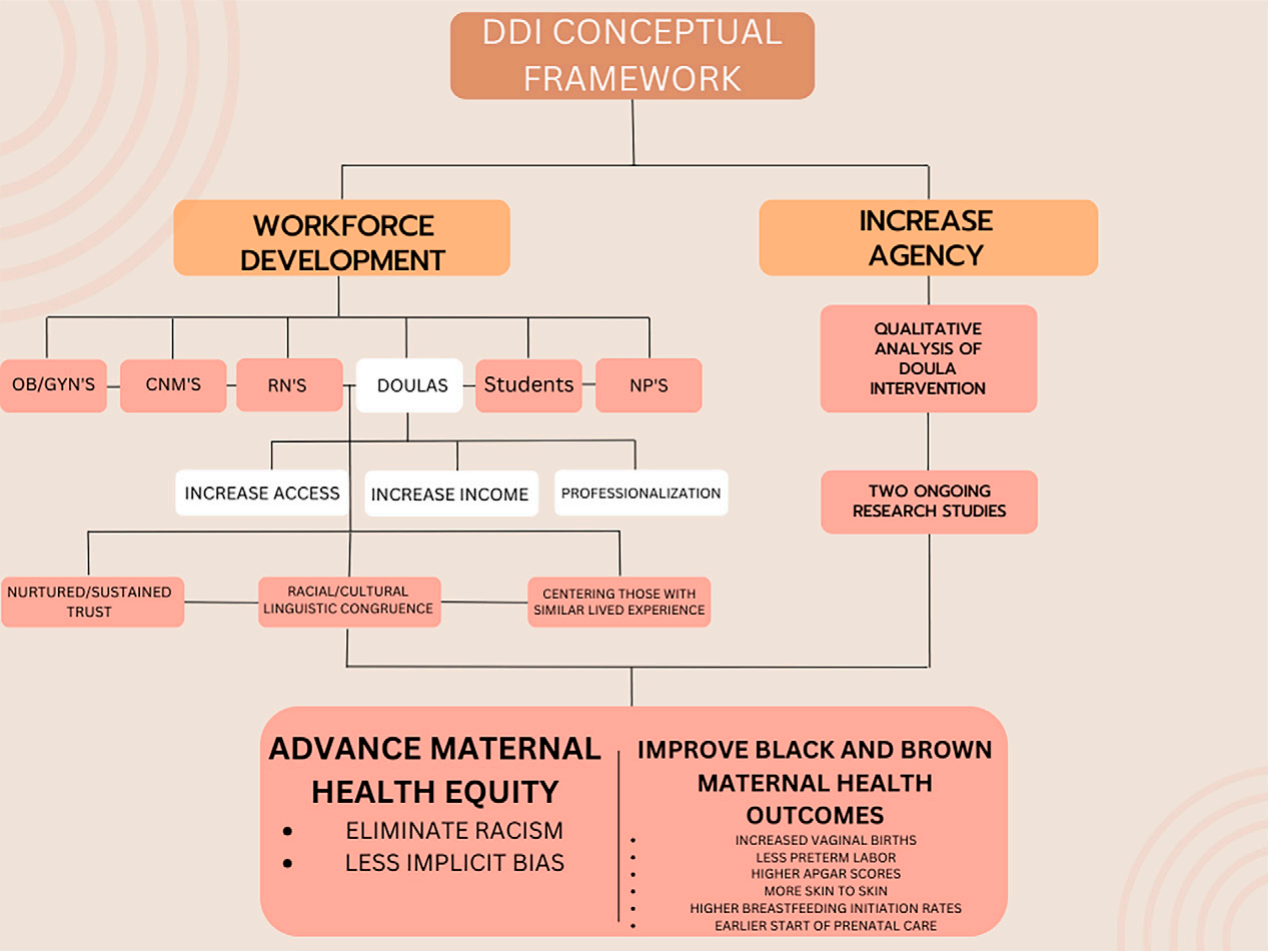
DDI Conceptual Framework. Improving patient birth experiences and strengthening the perinatal workforce can lead to improvement in Black and Brown maternal health outcomes and advance maternal health equity. CN, certified nurse midwife; RN, registered nurse; NP, nurse practitioners.

The DDI conceptual framework is grounded in prior evidence that maternal health outcomes are improved when doula care is incorporated into maternal care (Fig. 1).^4^ The intersection of doulas and obstetricians in DDI advances maternal health equity by emphasizing critical factors that lead to enhanced maternal health outcomes. Factors such as nurtured and sustained trust, racial/cultural/linguistic congruence, and centering those with similar lived experiences help promote this dyad as a vital part of improving Black and Brown maternal health outcomes. In addition, maternal health outcomes measured by the perinatal quality collaborative like substance use, severe hypertension, and postpartum depression are being addressed.^5^

According to the Pennsylvania Department of Health, in 2019, there were a total of 6,894 live births in Lancaster County. Of these live births, 335 (4.8%) were Black births, and 1,022 (14.8%) were Hispanic/Latinx births.^6^ From 2013 to 2018, PA had 547 pregnancy-associated deaths. While White mothers made up 70% of the births and 65% of the deaths during this time, Black mothers made up only 14% of births, yet 23% of deaths.^7^ With these statistics, it is important that we provide specific interventions that intend to positively impact obstetric outcomes in pregnant people of color.

## Program Design, Implementation, and Methods

The DDI team is made up of two board certified Obstetrics/Gynecology (Ob/Gyn) physicians. We recruited a certified nurse midwife, two certified doulas (CD), and two public health students that we worked with at our institutions. All team members had previous experience in the community birth work setting. We use a matrix on a Google cloud-based platform to store intake forms, contracts, invoices, and client-doula surveys. This information includes and monitors: patient consents (document created by the DDI Ob/Gyn physicians obtaining permission to share birth data with the DDI team), patient demographics, patient intake forms, patient-doula contracts, our doula provider list, doula training progress tracking sheets, finances, pre- and post-birth surveys, program workflow algorithm and policies, staff roster, meeting agendas, and minutes. The maternal health outcomes data being collected includes gestational age at time of birth, type of birth, incidence of hypertension, diabetes, depression, and substance use, breastfeeding rates at time of discharge and 4 weeks postpartum, and overall satisfaction with birth experience.

The purpose of collecting data is for quality improvement. We aim to measure success and satisfaction by comparing program participation levels against annual objectives and levels from the previous years of the program. For example, at UPMC Magee Women’s Hospital Birth Circles Doula program, an affiliate of one of the Lancaster hospitals, regardless of race or insurance, for every 100 deliveries under doula care, UPMC has come to expect 3–4 less preterm births, 5–7 more postpartum office visits, 7–11 more patients exclusively breastfeeding at discharge, and 13–24 more vaginal births after cesareans.^8^

Pre- and post-birth surveys are distributed to our clients and doulas. The surveys, created by DDI OB/Gyn board-certified physicians, consist of standard American College of Obstetricians and Gynecologists (ACOG) obstetric and birth data as guidelines. We obtain information tracking from both clients and doulas in terms of their own experiences, capacity, self-efficacy, knowledge, attitude, practice, and empowerment. Through our program matrix, qualitative and quantitative data are monitored and tracked including outcomes such as client demographics, prenatal and postpartum care, route of birth, gestational age at birth, birth location, infant outcomes, infant birth weight, and breastfeeding. We also tracked satisfaction of the DDI program by both the doulas and clients using pre and post care surveys. The goal is to assess both the client’s and doula’s satisfaction with the DDI program. The data will be used to evaluate the program’s effectiveness and provide information on how the program can improve its delivery of services and quality of care.

## Program Recruitment

DDI comprises recruitment, training, education, leadership development, and entrepreneurship for people of color who have an interest in maternal health. Doulas were recruited via social media and local marketing and advertising. Requirements to be a doula included: (1) identify as a person of color, (2) home address in Lancaster County, and (3) interest in maternal health. The application process was identical to applying for a traditional job or trade school. All applicants were required to send in a resume and cover letter to the official program email address. We aimed to remove any barriers that could potentially prevent doulas from applying. Once applications were submitted and reviewed, applicants were selected for a 30-minute interview. Questions regarding the individual’s background, skills, and interests were asked during the interview. After the interviews, applicants were selected for our doula training and education programs. Over 60 individuals expressed interest in the DDI program.

The doulas were evaluated on five components to determine their entrepreneurial goals: (1) quality of work history, (2) workload and skills, (3) financial incentive and marketing experience, (4) personal education and training, and (5) professional development. The components evaluated ensure that the doulas being trained can provide quality care, are committed to building their doula career, and support the longevity of the DDI program.

Our program implementation included training, delivery of care, and community activities. Training consisted of an 8-week online experience covering topics from reproductive anatomy to a “Birthing While Black” series.^9^ Trainees had access to and completed a 16-module doula school with certified doula trainers and a 12-month subscription to *Evidence Based Birth Practices.*^10^ Upon completion of these requirements, our doulas completed a 3-day in-person training with a certified DONA (Doulas of North America) International doula trainer. Because DDI was a new training organization, we chose DONA International to certify the DDI doulas since they are the first and largest doula training and certification organization. The doulas learned comfort measures, childbirth education, signs of labor, and more practices to help their clients’ birthing experiences. The trainees were divided into three cohorts. Each cohort received the 8-week online training and 3-day hands-on course. There were 8 trainees in the first cohort, 10 in the second cohort, and 10 in the third cohort. All three trainings took place over the course of 1 year. Delivery of services involving our trained and CD providing full scope doula care consists of two prenatal visits, birth support, and up to two postpartum visits.

## Results

From 2020 to 2022, we trained 28 doulas of color in Lancaster County. Prior to the creation of the DDI, Lancaster County had one Black doula. This represents a significant increase in doulas providing full scope doula care in Lancaster County. The DDI doulas and clients represent varying backgrounds, including African American, Caribbean, Latinx, Hispanic, and Middle Eastern.

**FIG. 2.**
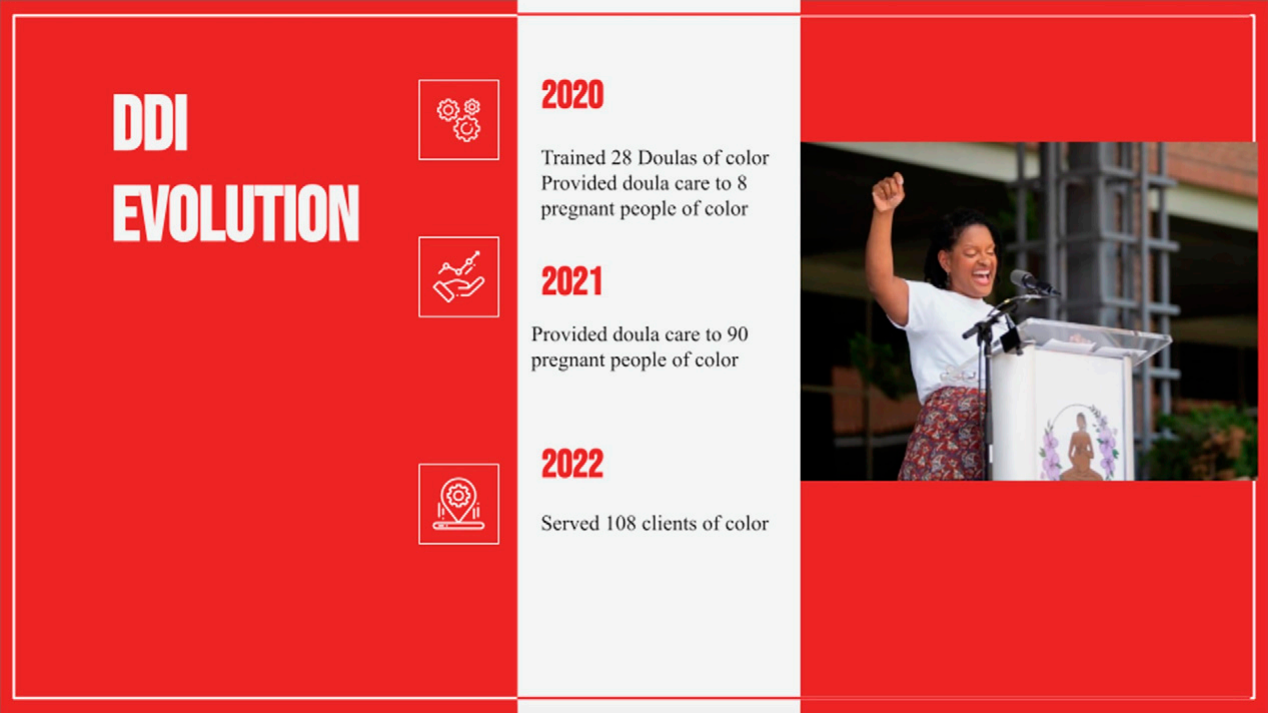
DDI held a community celebration in Downtown Lancaster to honor our second year of providing doula care.

Beginning in July 2020, DDI provided eight pregnant people of color with fully subsidized doula care. This number increased to 90 in 2021 and 108 in 2022 (Fig. 2).

## Discussion

DDI exists to increase access to doula care for underserved communities. In addition, we want to encourage other communities to create similar programs based on our success. Listening to the voices of those who make up DDI will undoubtedly enhance our growth.

DDI builds upon qualitative methods to better understand the first-hand experiences of both doulas and their clients. We strive to learn more about the environments of doulas and their clients to gain clarity on the client’s behaviors. This data can be enhanced with hospital-level data from the electronic medical record. In the future, we plan to examine other health indicators such as early initiation of prenatal care using the APNCU (Adequacy of Prenatal Care Utilization) index, intention to breastfeed, reproductive life plan, blood loss during labor, anemia, and pre-eclampsia. In addition, we will consider additional factors beyond race/ethnicity, such as zip code (geospatial residential clustering) and length of time in the U.S. (if not native-born). For future studies, DDI can determine program satisfaction by other stakeholders, such as nurses, midwives, students, and obstetricians who work alongside doulas. DDI designed two research studies to examine the success of our doula intervention.

## Strengths

The DDI is one of a kind. Through our research, we learned that no organized program like DDI exists. Two Black OB/Gyn physicians lead DDI. DDI is committed to increasing the number of Black and Brown doulas that provide fully subsidized care to birthing people of color. We are committed to diversifying the perinatal workforce and enhancing the relationship between obstetricians and doulas. Future directives could point to the need for expanding out “full scope doula care” to make a case for the need for longitudinal support, including inter-conception care, instead of being limited to only prenatal, intrapartum, and postpartum doula care.

## Limitations

Potential barriers that may arise during our program are doula retention and program sustainability. Many well-described benefits of cultural congruence exist in health care. Patients are more likely to adhere to preventive care and treatment plans if cared for by a provider with similar lived experiences. On the contrary, our doulas often encounter lived experiences identical to those of the clients they are serving. This juxtaposition can lead to supportive needs on both sides. DDI plans to create mechanisms for garnering longitudinal follow-up on the doula’s sustained professional growth and development.

## Conclusion

As a Black Ob/Gyn who values doulas, I strive to center the lives of marginalized birthing people and doulas. By valuing the lives of Black and Brown birthing people, dismantling systemic racism, and diversifying the perinatal workforce, we can bring back joy and happiness to Black and Brown birth and make Lancaster County the safest place to give birth.

## Abbreviation Used

ACOG: American College of Obstetricians and Gynecologists
APNCU: Adequacy of Prenatal Care Utilization
CD: Certified Doula
C.H.: Cherise Hamblin
C. J.-A.: Crista Johnson Agbakwu
CNM: Certified nurse midwife
DDI: Diversifying Doulas Initiative
DONA: Doulas of North America
K.B.: Kayla Bolden
LS. C.: LaShekia Chatman
NP: Nurse practitioner
Ob/Gyn: Obstetrician and Gynecologist
RN: Registered nurse
UPMC: University of Pittsburgh Medical Center
